# The Role of ZntA in Klebsiella pneumoniae Zinc Homeostasis

**DOI:** 10.1128/spectrum.01773-21

**Published:** 2022-01-12

**Authors:** Eve A. Maunders, Katherine Ganio, Andrew J. Hayes, Stephanie L. Neville, Mark R. Davies, Richard A. Strugnell, Christopher A. McDevitt, Aimee Tan

**Affiliations:** a Department of Microbiology and Immunology, The Peter Doherty Institute for Infection and Immunity, The University of Melbourne, Melbourne, Victoria, Australia; University of Hong Kong

**Keywords:** Klebsiella pneumoniae, zinc, metal transport, ATPase, zinc homeostasis, ZntA, metal biology

## Abstract

Klebsiella pneumoniae is an opportunistic Gram-negative pathogen that is a leading cause of healthcare-associated infections, including pneumonia, urinary tract infections, and sepsis. Essential to the colonization and infection by K. pneumoniae is the acquisition of nutrients, such as the transition metal ion zinc. Zinc has crucial structural and catalytic roles in the proteome of all organisms. Nevertheless, in excess, it has the potential to mediate significant toxicity by dysregulating the homeostasis of other transition elements, disrupting enzymatic processes, and perturbing metalloprotein cofactor acquisition. Here, we sought to elucidate the zinc detoxification mechanisms of K. pneumoniae, which remain poorly defined. Using the representative K. pneumoniae AJ218 strain, we showed that the P-type ATPase, ZntA, which is upregulated in response to cellular zinc stress, was the primary zinc efflux pathway. Deletion of *zntA* rendered K. pneumoniae AJ218 highly susceptible to exogenous zinc stress and manifested as an impaired growth phenotype and increased cellular accumulation of the metal. Loss of *zntA* also increased sensitivity to cadmium stress, indicating a role for this efflux pathway in cadmium resistance. Disruption of zinc homeostasis in the K. pneumoniae AJ218 Δ*zntA* strain also impacted manganese and iron homeostasis and was associated with increased production of biofilm. Collectively, this work showed the critical role of ZntA in K. pneumoniae zinc tolerance and provided a foundation for further studies on zinc homeostasis and the future development of novel antimicrobials to target this pathway.

**IMPORTANCE** Klebsiella pneumoniae is a leading cause of healthcare-associated infections, including pneumonia, urinary tract infections, and sepsis. Treatment of K. pneumoniae infections is becoming increasingly challenging due to high levels of antibiotic resistance and the rising prevalence of carbapenem-resistant, extended-spectrum β-lactamases producing strains. Zinc is essential to the colonization and infection by many bacterial pathogens but toxic in excess. This work described the first dissection of the pathways associated with resisting extracellular zinc stress in K. pneumoniae. This study revealed that the P-type ATPase ZntA was highly upregulated in response to exogenous zinc stress and played a major role in maintaining bacterial metal homeostasis. Knowledge of how this major bacterial pathogen resists zinc stress provided a foundation for antimicrobial development studies to target and abrogate their essential function.

## INTRODUCTION

Klebsiella pneumoniae is a ubiquitous environmental bacterium that asymptomatically colonizes human mucosal surfaces, including the intestinal tract. However, it is also a pathogen of significant concern and a causative agent of severe diseases in health care settings, such as pneumonia, urinary tract, and bloodstream infections (1, 2). Treatment of K. pneumoniae is becoming increasingly challenging due to high levels of circulating antibiotic resistance and the rising prevalence of carbapenem-resistant, extended-spectrum β-lactamases (ESBL) producing strains. Accordingly, the World Health Organization (WHO) has identified K. pneumoniae as a priority for the development of new antimicrobial therapies (3). Given the waning antimicrobial development pipeline and the lack of a vaccine, novel treatments and/or strategies for the control of multidrug-resistant infections are required.

First-row transition metals are essential to all forms of life. However, in excess, they can also mediate significant toxicity. In bacteria, the transition element zinc is estimated to provide structural stability and/or catalytic function for up to 6% of the bacterial proteome and contributes to diverse processes, such as the assembly of RNA polymerase and regulating zinc-finger protein functions (4–7). Despite the essentiality of zinc, its intracellular concentration must be tightly regulated to prevent inappropriate interaction with noncognate proteins. Intoxication studies have shown that zinc can compete with other metal cofactors for interaction with metalloproteins and disrupt crucial cellular processes, such as respiratory electron transport, homeostasis of other elements, metabolic pathways, membrane biogenesis, and cell morphology (8–10). During infection, host manipulation of elemental bioavailability contributes to the innate immune response with zinc intoxication occurring within the phagolysosome of neutrophils and macrophages and contributing to the efficacy of host control of infection (11).

Zinc homeostasis in bacteria is primarily dictated by zinc-responsive metalloregulatory proteins that control the expression of the import and export machinery and associated accessory proteins. In Escherichia coli, studies of the zinc metalloregulatory proteins Zur and ZntR indicated that sensing of cytoplasmic zinc occurred over an extremely narrow dynamic range with femtomolar sensitivity (12). Excess zinc in E. coli is primarily exported via two efflux systems, the P_1B_-type ATPase ZntA, and the cation diffusion facilitator (CDF) ZitB (4). Other membrane transport systems and chaperones have also been associated with zinc homeostasis, such as the zinc and iron-binding CDF transporter FieF (also known as YiiP) (13), but their contributions remain less well defined. This is highlighted by the prominent role of ZntA in E. coli survival during exposure to high zinc concentrations (14, 15). In contrast, deletion of ZitB, and/or other systems, such as FieF, have not been shown to significantly alter E. coli zinc resistance (14). The current paradigm for E. coli zinc tolerance is that ZitB serves as a constitutively expressed export pathway that contributes to zinc homeostasis during exposure to low to moderate levels of zinc. During exposure to high zinc concentration, the expression of *zntA* is upregulated with the P-type ATPase serving as a rapid and efficacious zinc exporter (15). In addition to these exporters, ZntB, a CorA-family protein first identified in S. enterica serovar Typhimurium (16), has also been implicated in zinc transport. Recent studies of E. coli ZntB (formerly YdaN) indicate a role in zinc import rather than export (17), although its precise contribution to zinc homeostasis remains to be fully defined.

In this study, we elucidated the poorly defined pathways used by K. pneumoniae to export excess zinc from the cell and achieve zinc tolerance. The data show that zinc is primarily exported from the bacterial cytoplasm by a homolog of the E. coli ZntA ATPase. Disruption of this pathway perturbs K. pneumoniae metal ion homeostasis, increases susceptibility to metal intoxication, and is associated with increased biofilm production. These data have implications for strategies to leverage bacterial susceptibility to metal stress in the development of antimicrobial therapies.

## RESULTS AND DISCUSSION

### ZntA is the primary exporter of zinc for K. pneumoniae.

**FIG 1:**
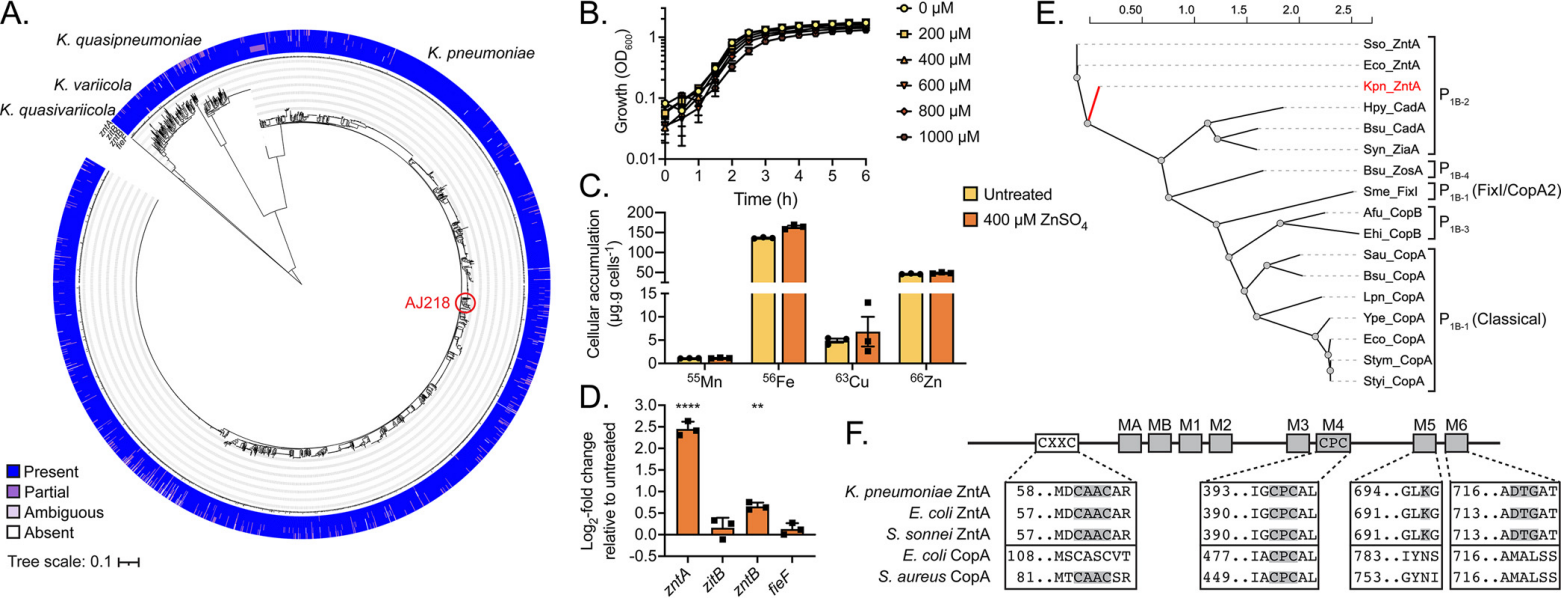
Zinc efflux mechanisms in K. pneumoniae. (A) Maximum likelihood phylogenetic tree of 2,706 publicly available Klebsiella spp. genomes with outer rings denoting the presence of AJ218 *zntA*, *zitB*, *zntB*, and *fieF* (outwards-in) as defined in Table S1. The position of AJ218 is indicated by a red circle. (B) Growth phenotype of K. pneumoniae AJ218 analyzed by optical density at 600 nm (OD_600_) in increasing ZnSO_4_ concentrations. Data are mean OD_600_ values (±SEM) from three (*n* = 3) independent biological experiments. (C) Cellular metal content (µg/g cells [dry weight]) of K. pneumoniae AJ218 grown in 400 μM ZnSO_4_ (*n* = 3, ±SEM). (D) qRT-PCR analysis of K. pneumoniae AJ218 grown in the presence or absence of 400 μM ZnSO_4_, corrected to untreated levels following internal normalization to *rpoD*. Data are mean values (±SEM) from three (*n* = 3) independent biological experiments. Statistical significance determined by 2-way ANOVA with Sidak posttest; **, *P* < 0.01; ****, *P* < 0.0001. (E) Phylogenetic analysis of P_1B_-type ATPases with subfamily groups shown. Functionally characterized proteins were used for analyses; Shigella sonnei, Sso (Q3YW59), E. coli, Eco (P37617 and Q59385), K. pneumoniae, Kpn (this study), Helicobacter pylori, Hpy (Q59465), Bacillus subtilis, Bsu (O32219, O32220, and O31688), *Synechocystis* sp. PCC 6803, Syn (Q59998), Sinorhizobium meliloti, Sme (P18398), Archaeoglobus fulgidus, Afu (O30085), Enterococcus hirae, Ehi (P05425), Staphylococcus aureus, Sau (Q2FV64), Legionella pneumophila, Lpn (Q5ZWR1), Yersinia pestis, Ype (Q8ZCA7), Salmonella enterica serovar Typhimurium, Stym (Q8ZR95), Salmonella typhi, Styi (Q8Z8S4). (F) Conservation of CXXC and CPC zinc/copper metal-binding motifs, and M5 and M6 transmembrane domain zinc ion coordinating residues in K. pneumoniae ZntA and representative ZntA and CopA proteins. Gray boxes represent conserved transmembrane domains (MA, MB, and M1-6) with conserved motif residues highlighted.

To elucidate the pathways associated with zinc tolerance in K. pneumoniae, the AJ218 genome was analyzed for putative transport systems with homology to known E. coli K-12 MG1655 (accession number U00096.3) zinc export pathways. This revealed K. pneumoniae homologues to the P-type ATPase, ZntA (76.9% identity, K. pneumoniae protein WP_048269565.1), the CorA-type metal ion transporter, ZntB (83.8% identity, WP_004148192.1), and the CDF family transporters ZitB (77.8% identity, WP_004147641.1) and FieF (89.3% identity, WP_002882907.1). Examination of 2,706 publicly available Klebsiella spp. genomes (18) revealed that all the putative zinc efflux genes were highly conserved in the Klebsiella population (Fig. 1A). All four genes were carried in >99.6% of genomes analyzed with high sequence conservation of present genes exceeding 99.4% pairwise identity (Table S1). Taken together, these data show that the putative zinc export pathways are highly conserved in the global Klebsiella spp. population and indicate that K. pneumoniae AJ218 is a suitable representative model for investigation of Klebsiella spp. zinc homeostasis.

The contribution of these systems to K. pneumoniae zinc tolerance was investigated using a combination of molecular and phenotypic approaches. Supplementation of Luria Bertani (LB) broth with increasing concentrations of ZnSO_4_ (0 to 1000 µM) had no discernible effect on the growth phenotype of K. pneumoniae AJ218 (Fig. 1B). Whole-cell accumulation of zinc in K. pneumoniae AJ218, determined by inductively coupled plasma mass spectrometry (ICP-MS), revealed no significant differences between cultures grown in 0 µM and 400 µM ZnSO_4_-supplemented media (Fig. 1C). Despite the lack of a discernible phenotypic impact, quantitative real-time PCR (qRT-PCR) revealed that *zntA* transcription was upregulated 5.7-fold in 400 µM ZnSO_4_-supplemented LB relative to the nonsupplemented medium. In contrast, no significant changes (<2-fold induction, *P* > 0.05) in the expression profiles of the other putative zinc transporter homologs, *zitB*, *zntB*, and *fieF*, were observed (Fig. 1D). Taken together, these data implicate ZntA as the primary K. pneumoniae AJ218 zinc efflux pathway expressed in response to zinc stress.

Bioinformatic analyses of K. pneumoniae ZntA indicated that it shared homology with P_1B_-type ATPases, which are associated with the transport of transition and heavy metal ions (19). Multiple sequence alignment and phylogenetic analysis of K. pneumoniae ZntA with other structurally or functionally characterized P_1B_-type ATPases revealed that ZntA clustered most closely with the type 2 P_1B_-type ATPases, notably with the zinc/cadmium-binding P_1B_-2-type ATPases Shigella sonnei ZntA (77.1% identity) and E. coli ZntA (76.9% identity) (Fig. 1E). Amino acid sequence comparisons revealed that functionally important regions, such as the CXXC and CPC zinc/copper metal-binding motifs, were conserved in K. pneumoniae ZntA (Fig. 1F). Moreover, K. pneumoniae ZntA also contains zinc ion coordinating residues within the M5 and M6 transmembrane domains, which are absent from copper-coordinating P_1B_-type ATPase CopA homologs, indicating a potential capacity for cadmium binding. Notably, ZntA-mediated cadmium export has been reported for E. coli and Salmonella typhimurium (20, 21). Accordingly, the contribution of ZntA to the homeostasis of zinc, cadmium, and other transition metal ions was investigated.

**FIG 2:**
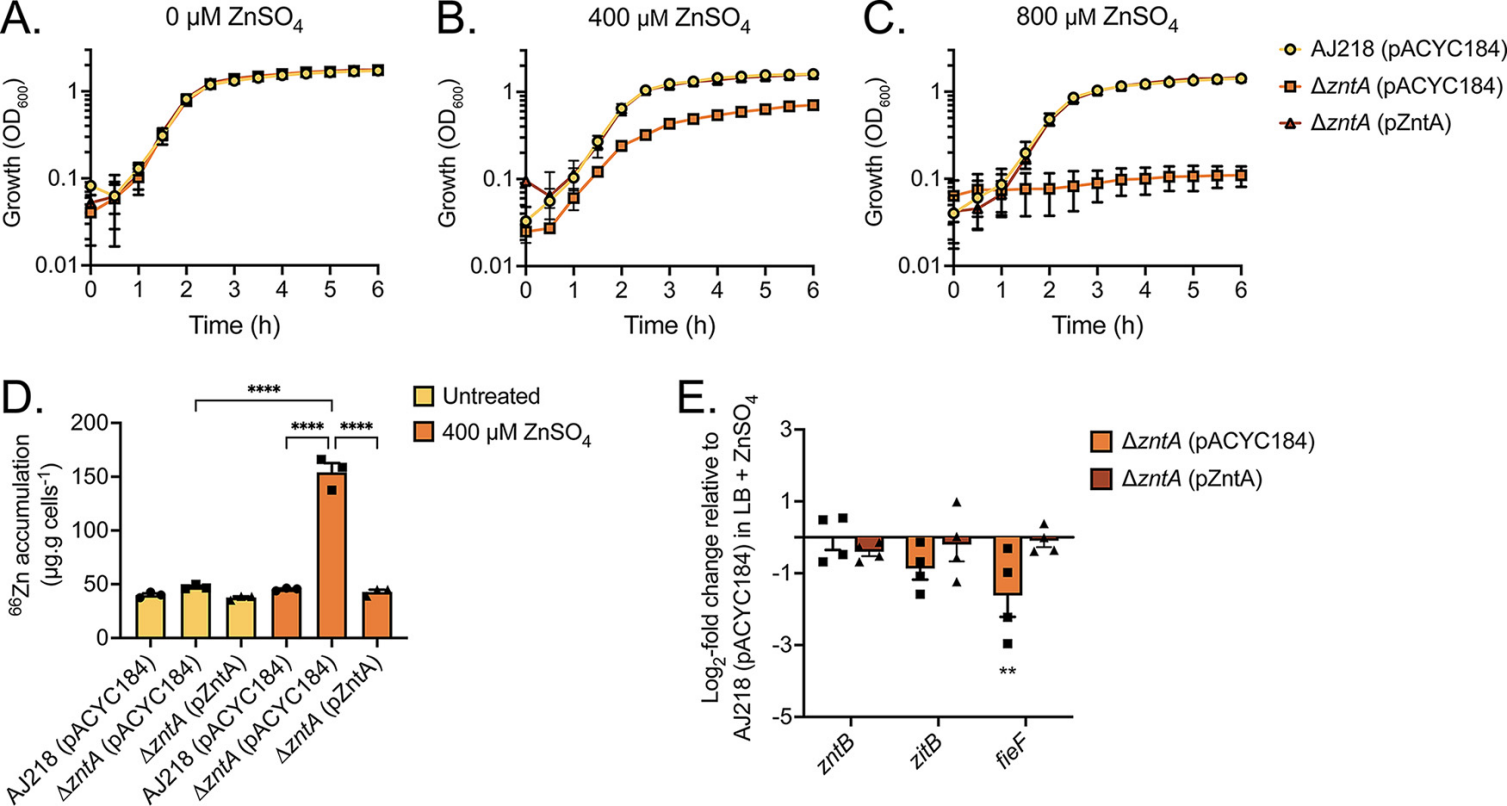
Characterization of K. pneumoniae AJ218 *ΔzntA.* Growth phenotypes of the wild-type, Δ*zntA*, and *trans-*complemented Δ*zntA* strains grown in (A) LB, and LB supplemented with (B) 400 μM ZnSO_4_, or (C) 800 μM ZnSO_4_. Data are mean OD_600_ values (±SEM) from three (*n* = 3) independent biological experiments. (D) The cellular zinc content of strains grown in LB with or without 400 µM ZnSO_4_ supplementation. Data are mean values (±SEM) from three (*n* = 3) independent biological experiments. Statistical significance of the differences determined by one-way ANOVA with Tukey posttest; ****, *P* < 0.0001 (E) qRT-PCR analysis of K. pneumoniae AJ218 wild-type, Δ*zntA* and the complemented strain grown in the presence of 400 μM zinc, corrected to wild-type levels following internal normalization to *rpoD*. Data are mean values (±SEM) from four (*n* = 4) independent biological experiments with statistical significance of the difference determined by 2-way ANOVA with Sidak posttest; **, *P* < 0.01.

To elucidate the contribution of ZntA to K. pneumoniae AJ218 zinc homeostasis, a *zntA* deletion mutant was generated, and this was complemented in *trans* on a low-copy-number plasmid with its native promoter (pACYC184::*zntA*; pZntA). The wild-type and derivative strains were assessed for their growth phenotype and cellular accumulation of metal ions in a zinc-supplemented LB medium. All strains showed comparable growth in a nonsupplemented LB medium (Fig. 2A). However, the Δ*zntA* strain showed perturbed growth in 400 μM ZnSO_4_ relative to the wild-type and complemented strain and abrogated growth at 800 μM ZnSO_4_ (Fig. 2B and C). Whole-cell metal accumulation of the K. pneumoniae strains revealed that the loss of *zntA* had no significant effect on cellular zinc in nonsupplemented media (Fig. 2D). Growth in 400 μM ZnSO_4_ supplemented LB resulted in a 3.4-fold increase in zinc accumulation in the Δ*zntA* mutant (*P* < 0.0001) relative to the wild-type and complemented strains (Fig. 2D).

The viability of Δ*zntA* in 400 µM ZnSO_4_ supplemented LB medium (Fig. 2B) suggested that other putative zinc export systems might compensate for the deletion of the P-type ATPase. Accordingly, qRT-PCR analysis of the putative zinc export systems was performed on K. pneumoniae AJ218 and the mutant derived strains grown in 400 µM supplemented ZnSO_4_ media. Analysis of the Δ*zntA* strain relative to the wild-type and complemented strains revealed that *zitB* and *zntB* were not significantly (*P* > 0.05) upregulated in the presence of exogenous zinc stress (Fig. 2E) while *fieF* was significantly downregulated (*P* < 0.05). Collectively, these data support a role for ZntA as a crucial zinc efflux pathway in K. pneumoniae AJ218. The lack of upregulation of the alternate putative zinc efflux transporters in K. pneumoniae suggested that they may not contribute in a significant manner to mitigating changes in bacterial zinc and/or their expression is uncoupled with cellular zinc abundance. Although *zitB* and *fieF* expression in response to zinc stress has been reported (14), the balance of evidence from recent studies suggests that *zitB* is constitutively expressed and does not respond to exogenous zinc abundance (15), while *fieF* expression is regulated by intracellular iron levels in a Fur-independent manner (13). Therefore, these data support a primary role for ZntA in responding to dynamic changes in K. pneumoniae zinc abundance. Although ZitB, ZntB, or FieF have the potential to contribute to Klebsiella zinc homeostasis, no definitive role(s) can be inferred based on the data arising from the experimental conditions investigated. Given the prominent contribution of ZntA to zinc homeostasis, its functional role was further investigated.

### ZntA contributes to cadmium homeostasis.

**FIG 3:**
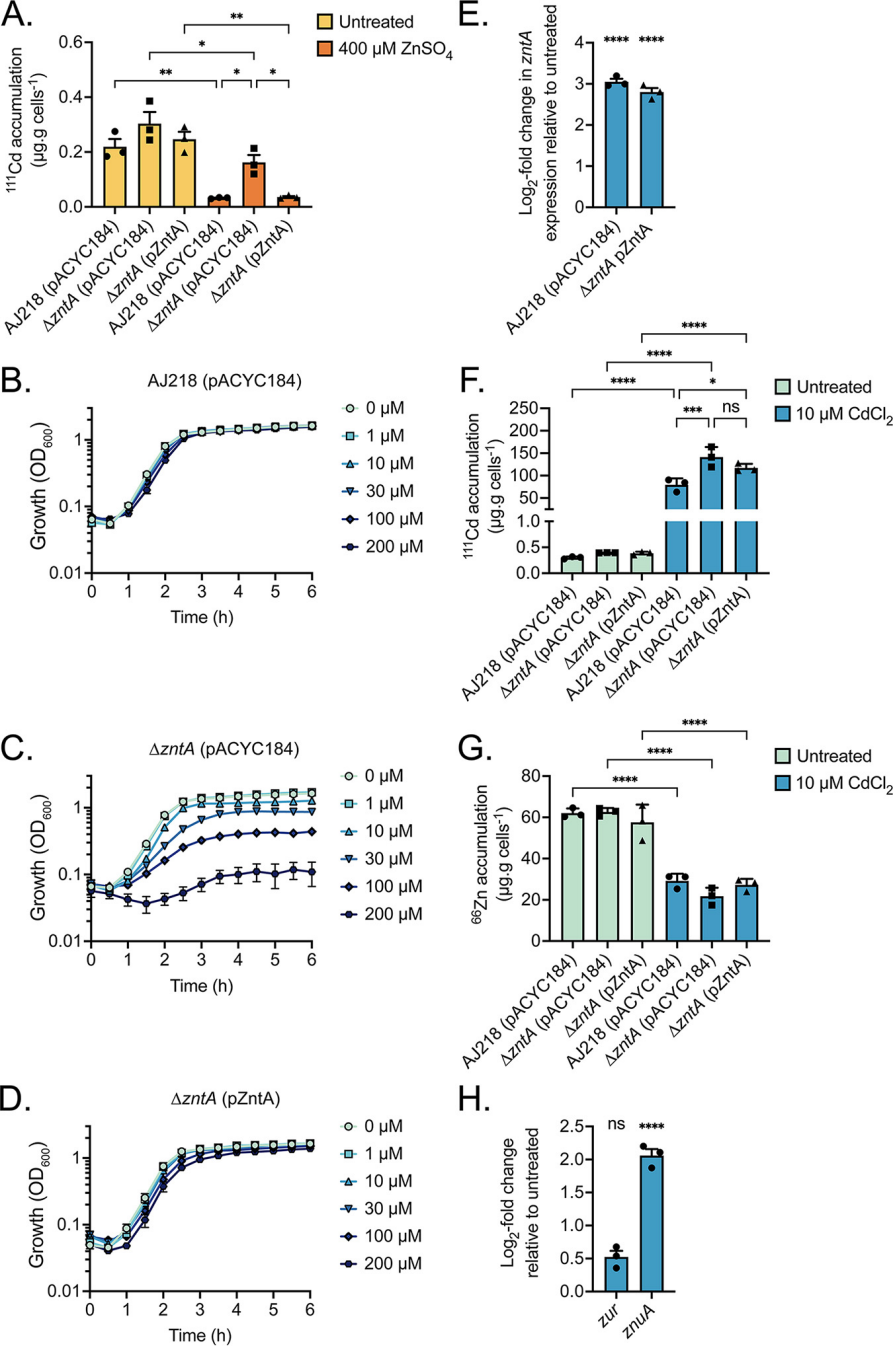
Role of ZntA in cadmium export. Cellular metal accumulation of K. pneumoniae AJ218 wild-type, Δ*zntA* and the complemented strains grown in LB supplemented with (A) 400 µM ZnSO_4_ or (F and G) 10 µM CdCl_2_. Data are mean values (±SEM) from three (*n* = 3) independent biological experiments with statistical significance of the differences determined by one-way ANOVA with Tukey posttest; ns, *P* > 0.05; *, *P* < 0.05; ** *P* < 0.01, ***, *P* < 0.001; and ****, *P* < 0.0001. Growth phenotypes of (B) K. pneumoniae AJ218 (pACYC184), (C) Δ*zntA* (pACYC184) and (D) the Δ*zntA* (pZntA) strains in increasing concentrations of CdCl_2_. Data are mean OD_600_ values (±SEM) from three (*n* = 3) independent biological experiments. qRT-PCR analysis of strains grown in 10 µM CdCl_2_ compared with untreated media, for *zntA* expression in wild-type and complement strains (E) and of putative *zur* and *znuA* genes in wild-type strain AJ218 (pACYC184) (H). Data are mean log_2_-fold change (±SEM, *n* = 3) relative to strains grown in untreated media with normalization to *rpoD.* Statistical significance of the differences determined by two-way ANOVA with Sidak posttest; ns, *P* > 0.05; ****, *P* < 0.0001.

Whole-cell metal accumulation revealed that K. pneumoniae strains grown in the zinc-supplemented medium had decreased cadmium accumulation by comparison to untreated cells (Fig. 3A). Notably, cadmium accumulation was ∼5.0-fold higher in the Δ*zntA* strain relative to the wild-type and complemented strains, which is consistent with a role for ZntA in cadmium efflux. To probe the contribution of ZntA to cadmium resistance, strains were grown in increasing CdCl_2_ concentrations (1 to 200 µM). This revealed that the Δ*zntA* strain was highly susceptible to cadmium stress relative to the wild-type and complemented strains (Fig. 3B to D). At a subinhibitory concentration of cadmium (10 µM CdCl_2_), the qRT-PCR analysis showed that *zntA* transcription was upregulated relative to untreated cells in wild-type and complement strains (*P* < 0.001, Fig. 3E). This shows that cadmium accumulation is associated with upregulation of *zntA* and is suggestive of a protective role against cadmium. However, cadmium-induced upregulation of *zntA* may also result in a concomitant perturbation of zinc homeostasis. Therefore, the impact of cadmium stress on metal ion homeostasis was investigated in wild-type and mutant derivative K. pneumoniae strains.

Supplementation of the growth medium with 10 µM CdCl_2_ increased cadmium levels in all strains (Fig. 3F). Accumulation of cadmium was higher in the Δ*zntA* strain relative to the wild-type but was not significantly different from the complemented strain. In contrast, zinc accumulation was significantly decreased in all strains upon exposure to cadmium to similar levels (Fig. 3G). The Δ*zntA* strain also showed reduced cellular zinc suggesting that upregulation of *zntA* in the wild-type and complemented strains (Fig. 3E) was not the primary cause of zinc depletion. It, therefore, follows that cadmium must be disrupting zinc import in K. pneumoniae AJ218. Accordingly, we investigated the hypothesis that cadmium ions could compete with zinc for import using a transcriptional approach. Expression of *znuA*, the gene encoding the putative solute-binding protein associated with the conserved bacterial zinc ATP-binding cassette permease ZnuABC, was monitored. Our data show that *znuA* was upregulated in K. pneumoniae AJ218 upon exposure to cadmium consistent with a transcriptional response to cellular zinc limitation (Fig. 3H). Taken together, the transcriptional analyses of *zntA* and *znuA* support a model wherein zinc accumulation is impaired by cadmium, at least in part, due to competition between the two metal ions for the zinc import pathway(s) of the bacterium. Further studies to elucidate the zinc import machinery of K. pneumoniae and the relative permissiveness of these pathway(s) to interact with cadmium ions are warranted.

### Loss of ZntA dysregulates the homeostasis of other transition metals.

**FIG 4:**
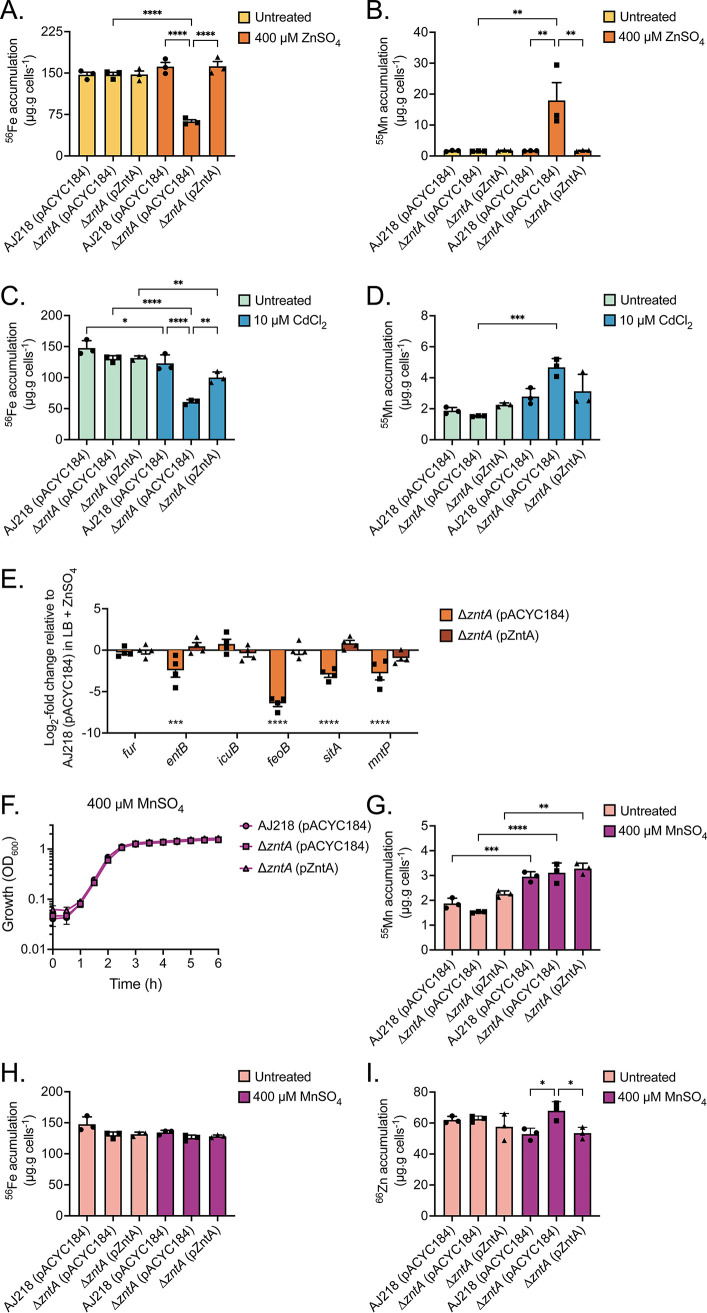
Impact of zinc and loss of *zntA* on cellular iron and manganese. Cellular accumulation of iron and manganese in K. pneumoniae AJ218 wild-type, Δ*zntA* and the complemented strains grown with or without 400 μM ZnSO_4_ (A and B) or 10 µM CdCl_2_ (C and D). Data are mean values (±SEM) from three (*n* = 3) independent biological experiments with statistical significance of the differences determined by one-way ANOVA with Tukey posttest; *, *P* < 0.05; **, *P* < 0.01; ***, *P* < 0.001; and ****, *P* < 0.0001. (E) qRT-PCR analysis of iron and manganese homeostasis associated genes in K. pneumoniae AJ218 Δ*zntA* and the complemented strain. Data are mean log_2_-fold change (±SEM) from four independent biological experiments (*n* = 4) relative to the wild-type strain grown in 400 μM ZnSO_4_ with normalization to *rpoD*. (F) Growth phenotype of the wild-type, Δ*zntA,* and the complemented strains in 400 µM MnSO_4_. Data are mean OD_600_ values (±SEM) from three (*n* = 3) independent biological experiments. Cellular accumulation of (G) manganese, (H) iron, and (I) zinc of K. pneumoniae strains grown in LB with or without 400 µM MnSO_4_ supplementation. Data are mean values (±SEM) from three (*n* = 3) independent biological experiments with statistical significance of the difference determined by one-way ANOVA with Tukey posttest; *, *P* < 0.05; **, *P* < 0.01; ***, *P* < 0.001; and ****, *P* < 0.0001.

The impact on the homeostasis of other transition metal ions was also investigated in the K. pneumoniae Δ*zntA* strain. During exposure to 400 µM ZnSO_4_, iron levels were significantly decreased (2.6-fold, *P* < 0.0001; Fig. 4A), consistent with the observed downregulation of *fieF*, while manganese levels significantly increased (10.9-fold; *P* < 0.01; Fig. 4B) in the Δ*zntA* strain relative to the wild-type and complemented strains. This pattern of dysregulated iron and manganese homeostasis was also observed in 10 µM CdCl_2_-treated K. pneumoniae Δ*zntA* relative to the wild-type and complemented strains (Fig. 4C and D). Notably, zinc stress did not perturb iron accumulation in wild-type AJ218 or the complemented strain (Fig. 4A), although cadmium treatment exerted a modest, but significant, iron depletion effect (Fig. 4C). Collectively, these data indicate that loss of ZntA renders K. pneumoniae susceptible to manganese and iron dyshomeostasis via exogenous zinc or cadmium stress.

To further investigate these dyshomeostasis impacts, the transcriptional profile of a selection of genes putatively associated with iron import (*fur*, *entB*, *icuB*, and *feoB*), and manganese uptake (*sitA*) and export (*mntP*), were analyzed in the K. pneumoniae strains exposed to 400 µM ZnSO_4_ (Fig. 4E). These results show that the enterobactin siderophore biosynthesis gene, *entB*, and ferrous iron uptake system, *feoB*, were significantly downregulated (*P* < 0.001) in the Δ*zntA* strain in the presence of extracellular zinc relative to the wild-type and complemented strains. The downregulation of these genes associated with iron uptake may be due to zinc-mismetallation of the ferric-uptake regulator Fur. However, this contrasts with E. coli, wherein increased zinc accumulation is associated with derepression of iron uptake genes and repression of iron-storage genes (8). This disparity may be explained by the observed increase in cellular manganese, also in contrast to what has been previously reported in E. coli (8). Previously it has been shown in E. coli and S. aureus that increased cellular manganese results in decreased iron levels (22, 23). This has been attributed to the manganese-based blockade of Fur activity. Thus, in K. pneumoniae Δ*zntA* the increased cellular quotient of manganese may be contributing to dysregulation of the cellular iron pools either directly or concomitantly with zinc. Elucidation of the precise molecular basis warrants further investigation.

K. pneumoniae strains grown in 400 µM ZnSO_4_ revealed that transcription of the manganese import (*sitA*) and efflux (*mntP*) pathways were both significantly downregulated (*P* < 0.0001; Fig. 4E) in the Δ*zntA* strain relative to the wild-type and complemented strains. Downregulation of both pathways may be due to zinc-mediated dysregulation of the manganese metalloregulatory control, which is comprised of MntR, Fur, and manganese-dependent riboswitch regulatory elements (24). This could arise from zinc-mediated repression of *mntP*, resulting in an increased level of cellular manganese. The increased abundance of manganese would then trigger repression of the *sitABCD* import pathway in a manganese-dependent manner. However, an alternative explanation may be that ZntA directly contributes to manganese homeostasis as an efflux pathway, which is similar to the P_1B_-type ATPase CtpC (25). To test that hypothesis, K. pneumoniae AJ218 and the mutant derivative strains were grown in increasing concentrations of MnSO_4_ (1 to 2000 µM) to investigate the potential for manganese toxicity (Fig. S1). No phenotypic growth impact was observed for any of the strains in response to manganese. Further analysis at the supraphysiological concentration of 400 µM MnSO_4_ showed no change in growth (Fig. 4F) and a modest increase in manganese relative to untreated cells in all strains (Fig. 4G). Importantly, the Δ*zntA* strain did not have significantly more manganese than the wild-type or complemented strains, and manganese levels were less than those observed for the zinc or cadmium stressed treatments (Fig. 4B and D). Taken together, these analyses indicate that ZntA does not serve as a manganese efflux pathway in K. pneumoniae. The increased manganese accumulation observed in the *ΔzntA* under zinc stress warrants further investigation in future studies. Accumulation of iron was unaffected by manganese stress in all strains (Fig. 4H). However, zinc levels were significantly increased in the Δ*zntA* strain relative to the wild-type and complemented strain in the presence of manganese (Fig. 4I). This may suggest that cellular manganese influences the expression of zinc uptake and/or efflux, but the extent of this cross-talk did not have an apparent physiological impact.

### Biofilm formation aids in resistance to zinc stress.

K. pneumoniae forms biofilms during infection of the respiratory, gastrointestinal, and urinary tracts (26). Biofilm formation is one mechanism by which some bacteria resist extracellular stress, such as heavy metal ions (27–29). Iron is established as an important trace element for Klebsiella biofilm formation (30, 31), while multiple studies have shown that cellular zinc is a critical cofactor for cell surface adhesins that contribute to the formation and stability of biofilms (32–34). Despite this, high concentrations of extracellular zinc have been shown to exert mild inhibitory effects on the biofilm formation capacity of urinary tract isolates of K. pneumoniae (35). It was hypothesized that this arose due to zinc-mediated inhibition of iron uptake via mismetallation of Fur, although direct evidence remains lacking. Therefore, the influence of zinc on K. pneumoniae AJ218 biofilm formation was investigated.

**FIG 5:**
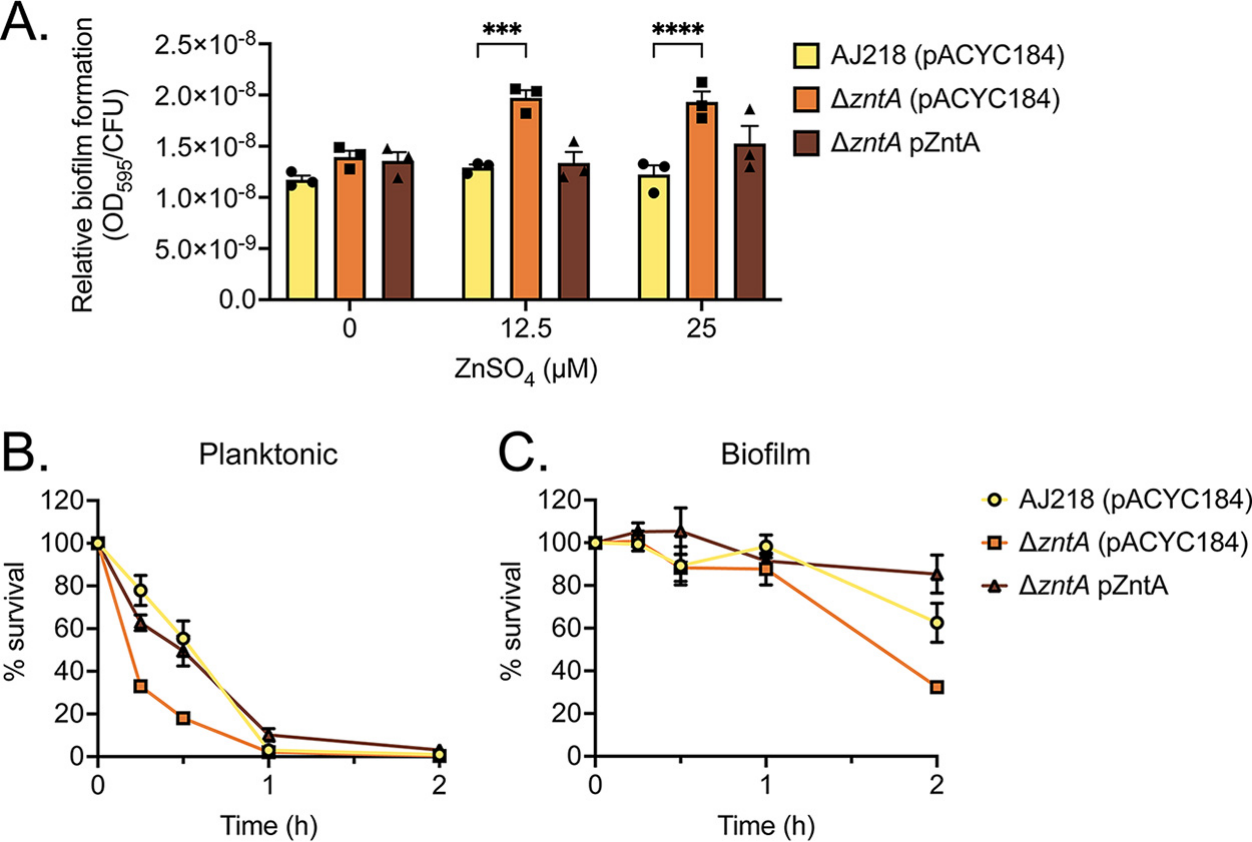
Biofilm formation and zinc resistance by K. pneumoniae AJ218, *ΔzntA*, and complemented strains. (A) Relative biofilm formation of K. pneumoniae AJ218 wild-type, Δ*zntA* and the complemented strains grown in minimal media with increasing concentrations of ZnSO_4_. Data are mean values (±SEM) from three (*n* = 3) independent biological samples of biofilm matrix quantified by crystal violet absorbance (595 nm) and normalized relative to CFU. Survival of planktonic (B) and biofilm (C) cultures of K. pneumoniae strains challenged with saline containing 800 µM ZnSO_4_. Data are mean bacterial survival (CFU.Ml^−1^ ± SEM), from three (*n* = 3) independent biological experiments relative to the untreated control, determined over 2 h posttreatment.

The K. pneumoniae AJ218 wild-type and derivative strains were grown in M9 minimal media supplemented with subinhibitory zinc concentrations (12.5 and 25 µM ZnSO_4_) in microtiter plates. The surface-adhered biomass was measured by crystal violet staining and standardized to the colony forming unit (CFU) within the biofilm to account for potential zinc stress-related impacts to cell density. At subinhibitory concentrations of zinc, the Δ*zntA* strain produced more biofilm, while the wild-type and complemented strains did not significantly change (Fig. 5A). These data indicate that the inability to efflux zinc efficiently leads to increased biofilm formation when excess zinc is present in the external milieu.

To ascertain whether biofilm formation was enhancing stress resistance, the relative susceptibility of the planktonic and biofilm K. pneumoniae cells to zinc stress was investigated. This was addressed by growing the cells in M9 minimal media, isolating biofilm and planktonic cells, and then challenging them with 800 µM ZnSO_4_ and measuring survival over 2 h of the challenge. This revealed that all strains had comparable survival of biofilm cells over the first 1 h of challenge with only the Δ*zntA* strain showing a lower survival by 2 h, by comparison to the wild-type and complemented strains. The planktonic cells of all strains showed reduced survival to zinc challenge relative to the biofilm-forming cells with less than 10% survival by 1 h (Fig. 5B and C). Notably, the planktonic Δ*zntA* strain succumbed to zinc stress more rapidly than the wild-type or complemented strains (Fig. 5B). These data suggest that K. pneumoniae AJ218 biofilm formation contributes to resistance against zinc stress. This could arise from the biofilm matrix components chelating or slowing permeation of zinc ion diffusion and thereby protecting K. pneumoniae. In particular, extracellular DNA (eDNA), which is a major constituent of biofilm matrices, has known cation-chelating properties (36).

Collectively, this work elucidates the primacy of ZntA as the major efflux pathway for zinc and cadmium detoxification in K. pneumoniae. Our data highlights that strict regulation of zinc homeostasis is critical to prevent dyshomeostasis of iron and manganese, suggesting that zinc intoxication has the potential to disrupt other cellular processes. Resistance to zinc intoxication can be enhanced by the formation of biofilm, which suggests that components of the biofilm matrix may act to buffer or sequester metal ions. These insights advance our understanding of the molecular mechanisms of K. pneumoniae zinc homeostasis, which may serve as potential targets in future antimicrobial development approaches.

## MATERIALS AND METHODS

### Bioinformatic analyses.

Putative K. pneumoniae AJ218 zinc resistance mechanisms were identified by BLASTP alignment to E. coli K-12 MG1655 candidates. Maximum likelihood phylogenetic analyses of the P_1B_-type ATPases were performed through the web server NGPhylogeny workflow using MAFFT, BMGE, and PhyML software following the LG substitution model (37). Branch statistics were calculated using 1000 bootstrap replications.

### Comparative genomics.

A database of 2,706 publicly available Klebsiella genomes and associated maximum likelihood phylogenetic tree (18) was screened for zinc homeostasis genes by the BLASTN screening tool, Screen Assembly (v1.2.7) (38), applying cutoffs of 80% identity and 80% reference length. To reduce the false-negative rate (e.g., due to contig breaks), gene absence was further validated by screening 300 bp segments of each target gene (3 segments for *zntB*, *zitB*, *fieF;* 5 for *zntA*; Table S1), and hits were mapped onto the phylogenetic tree using Interactive Tree Of Life (iTOL version 6.1) (39). Translated full-length protein sequences were used for variation analysis by MUSCLE Alignment (version 3.8.425) (40).

### Bacterial strains, chemicals, media, and growth.

Bacterial strains and plasmids used in this study are listed in Table 1. All strains were routinely cultured with Luria Bertani broth (LB). For growth assays, overnight cultures of K. pneumoniae were standardized (optical density at 600 nm (OD_600_) = 0.05) and incubated at 37°C with shaking in a FLUOStar Omega spectrophotometer. Media were supplemented, where appropriate, with kanamycin (Km) 50 μg/mL; chloramphenicol (Chl), 30 μg/mL for E. coli, and 80 μg/mL for K. pneumoniae; and ZnSO_4_, MnSO_4,_ or CdCl_2_ as stated in the text.

**TABLE 1:** Strains and plasmids used in this study

Strain or plasmid	Genotype or description	Source or reference
K. pneumoniae		
AJ218	Wild-type, clinical isolate, serotype K54; Ap^R^	(41)
Δ*zntA*	AJ218 deletion mutant *zntA*::*Km;* Ap^R^ Km^R^	This study
E. coli		
NEB 5-α	High efficiency competent E. coli, *fhuA2* (*argF-lacZ*)U169 *phoA glnV44* 80 (*lacZ*)M15 *gyrA96 recA1 relA1 endA1 thi-1 hsdR17*	New England Biolabs
		
Plasmids		
pACYC184	Low-copy-number cloning vector, p15A ori; Tet^R^ Chl^R^	New England Biolabs
pZntA	AJ218 *zntA* cloned into pACYC184; Chl^R^	This study
pKD4	Source of FRT-flanked Kan^R^ cassette; Ap^R^ Km^R^	(44)
pACBSR	Ara promoter control, I-SceI and λ Red recombinase; Chl^R^	(45)

### Construction of K. pneumoniae gene deletion mutants.

The *zntA* deletion mutant was generated by adapting the gene gorging method (41) as follows: using the NEBuilder HiFi DNA Assembly Master Mix (New England Biolabs, NEB) to flank a kanamycin resistance cassette from pKD4 with ∼500 bp upstream and downstream of the *zntA* gene. Two hundred nanograms of the linear construct were electroporated into electrocompetent K. pneumoniae AJ218 cells containing the plasmid pACBSR, encoding an l-arabinose inducible lambda Red recombinase gene. Complementation vectors were made by amplification of AJ218 *zntA* and 300 bp upstream, including its native promoter and insertion into the *tet* gene of pACYC184, using NEBuilder. All primers are listed in Table S2.

### Cellular metal ion content analysis.

Overnight K. pneumoniae cultures were standardized to an OD_600_ of 0.05 and grown in biological triplicate to mid-log-phase (OD_600_ = 0.8 to 1) in untreated and metal supplemented media. Cultures were washed twice with phosphate-buffered saline (PBS) containing 5 mM ethylenediaminetetraacetic acid, and twice with PBS. Bacterial pellets were desiccated at 95°C overnight then digested in 250 μL 65% (vol/vol) HNO_3_ at 95°C for 20 min. Samples were centrifuged at 18,000 × *g* for 25 min and soluble material was diluted to a final concentration of 3.25% HNO_3_ using MilliQ-H_2_O. Elemental content was quantitatively analyzed in technical triplicate using an Agilent 8900 ICP-QQQ-MS (Agilent Technologies).

### RNA isolation and qRT-PCR.

K. pneumoniae strains were grown to mid-log-phase (OD_600_ = 0.8 to 1) and harvested by centrifugation (18,000 × *g*, 5 min) and resuspended in RNA Protect Bacteria Reagent (Qiagen). Total RNA was extracted using the RNeasy minikit, digested twice with on-the-column DNase I digestion, as per the manufacturer’s guidelines. Samples were quantified using the FLUOStar Omega Spectrophotometer.

Quantitative real-time PCR was performed on the QuantStudio 7 real-time PCR system (Thermo Fisher Scientific) using the SuperScript III Platinum SYBR Green one-step qPCR Mix according to the manufacturer’s instructions. Oligonucleotides used for qRT-PCR are listed in Table S2. The gene *rpoD* was used to normalize gene expression. The data represent at least three biological replicates.

### Biofilm culture and quantification.

Biofilm cultures were grown in duplicate in 24-well microtiter plates. Bacterial cultures were normalized (OD_600_=0.05) in M9 minimal media (42) and statically incubated for 24 h at 37°C with ZnSO_4_ supplementation. After 24 h growth, planktonic cells were discarded, and biofilm cells were washed with PBS. For analysis, one replicate was used to determine CFU by a detachment of biofilm cells by pipetting into 1 mL PBS followed by serial dilution and viable count on LB agar. The other replicate was used to quantify biofilm, adapting the crystal violet stain method (43) with wash by aspiration. Biofilm mass was quantified by measuring absorbance (595 nm) relative to CFU.

### Zinc susceptibility assay.

Biofilm cultures were grown for 24 h in untreated media. From each well, 1 ml of planktonic culture was centrifuged at 18,000 × *g* for 10 min and the remaining biofilm was washed once with saline solution (0.85% NaCl). A saline solution containing 800 µM ZnSO_4_ was added directly onto the biofilm cells and used to resuspend the planktonic culture. The cells were incubated statically at 37°C and CFU/mL was determined by viable count. Relative survival was determined by comparison to an untreated saline condition.

### Statistical analysis.

Unless otherwise stated, the data represent the mean of biological triplicates (±standard error of the mean [SEM]). Statistical analyses were performed using a two-tailed Student's *t* test when comparing two data sets or a one-way ANOVA test for >2 data sets. Comparisons to the wild-type or untreated condition were realized by the Tukey or Sidak posttest as specified in figure legends; ns = not significant; *, *P* < 0.05; **, *P* < 0.01; ***, *P* < 0.001; and ****, *P* < 0.0001.
