# Supplementary material for: The Role of ZntA in Klebsiella pneumoniae Zinc Homeostasis
Source: Microbiol Spectr. 2022 Jan 12;10(1):e01773-21. doi: 10.1128/spectrum.01773-21 (PMC8754117; doi:10.1128/spectrum.01773-21)
Supplement: SUPPLEMENTAL FILE 2 — Supplemental material. Download SPECTRUM01773-21_Supp_2_seq3.pdf, PDF file, 0.3 MB [file spectrum01773-21_supp_2_seq3.pdf]

Supplementary Figure 1.

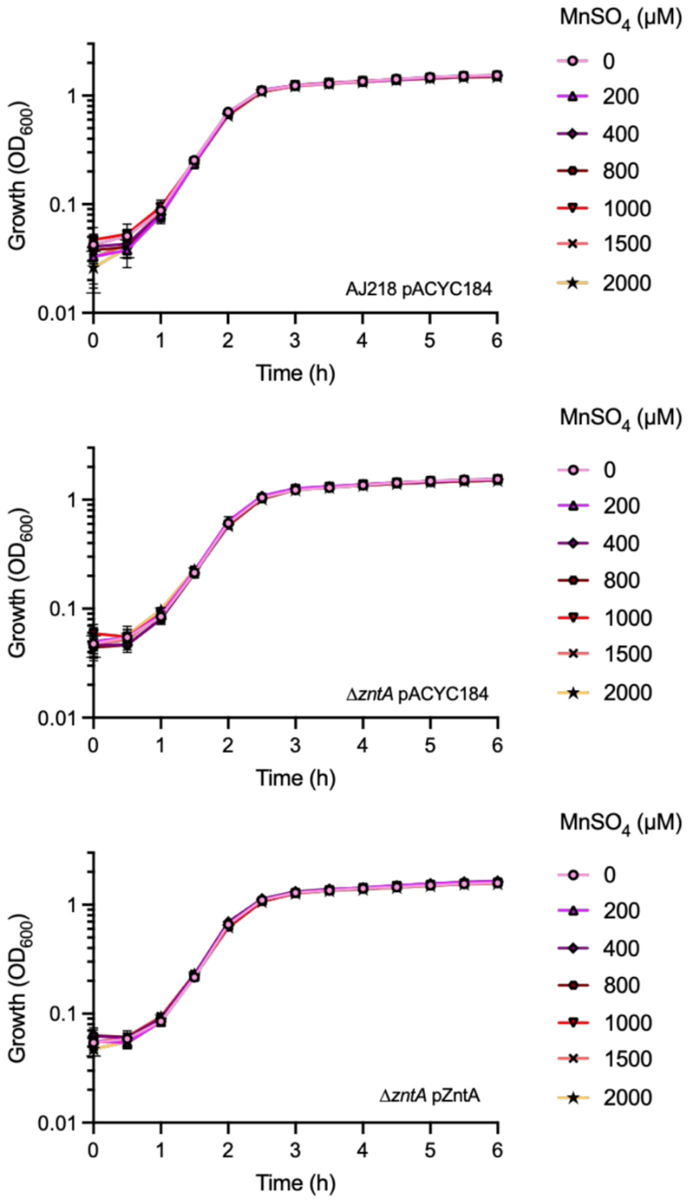

Figure S1. Impact of manganese on *K. pneumoniae* AJ218 wild-type and complemented strains.

Growth phenotypes of the AJ218 pACYC184 (top), Δ*zntA* (pACYC184) (middle) and Δ*zntA* pZntA (bottom) in the presence of increasing manganese (0 - 2000 μM). Data are representative mean OD_{600} values (±SEM; n=3).

Supplementary Table 2. Oligonucleotides used in this study.

**Primer name**	**Function**	**Sequence 5' – 3'**
**Gene deletions**		
*zntA*LF-F	Amplifies the *zntA* left flanking sequence	GGCGGCCGCGGGAATTCGATGCTTTCTTCCTGTCACATCTGC
*zntA*LF-R	Amplifies the *zntA* left flanking sequence incorporating overlapping *Km* sequences	AAGCAGCTCCAGCCTACACAGGCACCCTCCGGGAAATG
*zntA*RF-F	Amplifies the *zntA* right flanking sequence incorporating overlapping *Km* sequences	CTAAGGAGGATATTCATATGGCATAGATACCGGCTTCC
*zntA*RF-R	Amplifies the *zntA* right flanking sequence	GCCGCGAATTCACTAGTGATGTCATGCTTCAGGTGAATG
zntA-OUTF	Amplifies upstream of the allelic exchange *zntA* site	CTGTCTGTTTCTCCGCATGG
zntA-OUTR	Amplifies downstream of the allelic exchange *zntA* site	GAGATTAAAGGTGCCCAGCG
***Km*^{R} gene**		
pKD4F	Amplifies the *Km* resistance gene from pKD4	TGTGTAGGCTGGAGCTGCTTC
pKD4R		ATGAATATCCTCCTTAGTTCCTATTCC
pKD4Fseq	Amplifies outwards from the *Km* 3' sequence to confirm genomic gene deletion	TGACGAGTTCTTCTGAGCGGGAC
pKD4Rseq	Amplifies outwards from the *Km* 5' sequence to confirm genomic gene deletion	CAGTCTAGCTATCGCCATGTAAGCC
**Complementation constructs**		
pACYC184 F	Linearises the pACYC184 plasmid within the *tet* gene	ACGGGTGCGCATAGAAATTGC
pACYC184 R	Linearises the pACYC184 plasmid within the *tet* gene	GCGAGAAGCAGGCCATTATCG
*zntA*compF	Amplifies *zntA* gene and native promoter for assembly into pACYC184	GATAATGGCCTGCTTCTCGCATCGGAATGACGCTGGTAATG
*zntA*compR		CAATTTCTATGCGCACCCGTGCAGGGAAGCCGGTATCTATG
**qRT-PCR**		
zntA-F	Amplifies *zntA* gene	CAATTCTCGTCTTTCACCATGCG
zntA-R		GCCATCAACTTGCCAGCTATAG
zitB-F	Amplifies *zitB* gene	CCTGGCGCTTACTGAAAGAGAG
zitB-R		TGAACATCGCGTACCTCTGG
zntB-F	Amplifies *zntB* gene	GTATATGGATGAGCGGCTGATCG
zntB-R		CAACGCATCGCAGACCTCC
fieF-F	Amplifies *fieF* gene	GCTGGTGGATTCGCTGGTG
fieF-R		CGACTCCGCTTTGCCATGAC
fur-F	Amplifies *fur* gene	GCATTAAAGAAGGCTGGCC
fur-R		CTTCCGCACTGACATGATGG
entB-F	Amplifies *entB* gene	GATGATGGAGAAAGTGGTGG

entB-R		CGGTCTTCATCGCTCTGC
icuB-F	Amplifies *icuB* gene	GGAAACGCAGTGCTTAGTGG
icuB-R		GCCAGAAGCCATCAATAAAATC
feoB-F	Amplifies *feoB* gene	ATCCTAATTCCGGCAAGACC
feoB-R		GCGAAAATCCCCTCTTTACG
sitA-F	Amplifies *sitA* gene	GACGTGAGCTCCATTACTAAACC
sitA-R		CGTTCGAGATTAAGCCCATTGC
mntP-F	Amplifies *mntP* gene	GCTACCATTCTTCTCGCTTTCG
mntP-R		CGTACTGCCTCTGAAAATTTGGG
*rpoD*562F	Amplifies *rpoD* gene	GAAGAGATGGATGACGACGAAGACG
*rpoD*677R		GTACGCAGCTCGGCGAATTTCTCAC
znuA-F	Amplifies *znuA* gene	CGAGACCCAGGTTCTGCTG
znuA-R		CACACCACTAAGTCTGCGTTC
Zur_rt_F1	Amplifies *zur* gene	GTCTGGAAGTGTTGCGCCTG
Zur_rt_r1		CTCCAGCGCACGGTAAACC

Gene-specific sequences in gene deletion and complementation primers are underlined.
